# The impact of disease modifying therapies on cognitive functions typically impaired in multiple sclerosis patients: a clinician’s review

**DOI:** 10.3389/fneur.2023.1222574

**Published:** 2023-07-12

**Authors:** Karolina Kania, Wojciech Ambrosius, Wojciech Kozubski, Alicja Kalinowska-Łyszczarz

**Affiliations:** ^1^Department of Neurology, Poznan University of Medical Sciences, Poznań, Poland; ^2^Department of Neurology, Division of Neurochemistry and Neuropathology, Poznan University of Medical Sciences, Poznań, Poland

**Keywords:** cognitive functions, multiple sclerosis, disease modifying therapies, SDMT, brain atrophy

## Abstract

**Objective:**

Over the last few decades clinicians have become aware that cognitive impairment might be a major cause of disability, loss of employment and poor quality of life in patients suffering from multiple sclerosis [MS].

The impact of disease modifying therapies [DMTs] on cognition is still a matter of debate. Theoretically, DMTs could exert a substantial beneficial effect by means of reducing neuroinflammation and brain atrophy, which are established correlates of cognitive dysfunction. The aim of the study was to review the evidence concerning the effect of DMTs on cognitive functions.

**Methods:**

PubMed, Scopus, and the European Committee for Treatment and Research in Multiple Sclerosis [ECTRIMS] Library were searched for articles concerning the pediatric and adult populations of patients with multiple sclerosis, including clinical trials and RWD, where psychometric results were analyzed as secondary or exploratory endpoints.

**Results:**

We reviewed a total of 44 studies that were found by our search strategy, analyzed the psychological tests that were applied, the length of the follow-up, and possible limitations. We pointed out the difficulties associated with assessing of DMTs’ effects on cognitive functions, and pitfalls in cognitive tools used for evaluating of MS patients.

**Conclusion:**

There is a need to highlight this aspect of MS therapies, and to collect adequate data to make informed therapeutic decisions, to improve our understanding of MS-related cognitive dysfunction and provide new therapeutic targets.

## 1. Introduction

Multiple sclerosis [MS] is an autoimmune demyelinating disorder of the central nervous system [CNS], typically affecting the people between 20 and 40 years of age. It is considered one of the most common non-traumatic diseases of the brain leading to disability in young adults (1). Not only does MS cause physical disability, but also impairs cognition, the latter affecting patients’ quality of life even more profoundly. Cognitive dysfunction has a prevalence rate of approximately 50%, but for patients it is far from benign and remains highly relevant for daily functioning (2). The most vulnerable phase for the progression of cognitive deficits seems to occur during the first 5 years after disease onset (2). Moreover, the percentage of patients with cognitive decline is likely underestimated since detailed neuropsychological tests are not routinely assessed in clinical practice. Also, the fundamental clinimetric scale used to score MS patients’ disability, namely Expanded Disability Status Scale [EDSS], does not sufficiently reflect cognitive impairment. EDSS is influenced mainly by the assessment of physical disability, especially gait impairment. On the other hand, cognition is scored in EDSS as: normal, decrease in “mentation,” or dementia. In MS severe dementia syndromes with disorientation as to time and place are very rare (score 4 and 5), but score 2–without more accurate psychological testing - does not differentiate well within the largest group of patients with milder cognitive impairment (3). Consequently, it is difficult to establish either cognitive relapses or progressive cognitive decline in MS patients. While we do know cognitive relapses occur (4) they may not necessarily cause any increase in EDSS score, and thus might not be considered as evidence for treatment failure.

Cognitive impairment may be present since the earliest stages of the disease, even in radiologically isolated syndrome [RIS], where patients were shown to score below the mean performance for the healthy population on neuropsychological testing (5). In RIS subjects, the most common deficit was observed in information processing speed, similar to patients with clinically definite MS (5). Cognitive domains that are most severely affected in MS include: information processing speed, learning memory, executive functions and attention (2, 5, 6). More frequent and severe deficits are reported in secondary progressive MS [SPMS] and primary progressive MS [PPMS], especially in the working memory and executive functions domains, which can be associated with predominant gray matter pathology in the progressive stages of the disease (6). The impact of disease modifying therapies [DMTs] on cognition is still a matter of debate. Theoretically, DMTs could exert a substantial beneficial effect by means of reducing neuroinflammation and brain atrophy, which are established correlates of cognitive dysfunction. The aim of the study was to review the evidence concerning the effect of DMTs on cognitive functions and to emphasize on difficulties with the analysis of these functions, resulting from the still small number of studies in this field and lack of one, standard neuropsychological battery.

### 1.1. Clinical relevance of cognitive dysfunction and cognitive assessment tools in MS patients

Cognitive dysfunction is one of the major factors determining the quality of life in patients with MS. It was shown that cognitively impaired patients were more likely unemployed, experienced greater difficulty in performing household tasks and were more often socially withdrawn (7).

Unemployment is common in individuals with MS (7)Cognitive dysfunction impacts employment, and unfortunately EDSS does not predict employment status (8), whereas studies have shown that cognitive performance on SDMT is associated with earnings (9).

Importantly, the commonly used term of “benign MS” is defined as a long-term [> 10 years] disease course with EDSS ≤3, but this definition does not include cognitive functioning (10). However, in one study neuropsychological deficits have been documented in 45% of patients with “benign MS” (10). These patients exhibited significantly higher handicap scores and significant restrictions of their everyday activity (11).

The three most frequently used neuropsychological/psychometric batteries in MS are: [i] The Brief Repeatable Battery of Neuropsychological tests [BRB-N], also known as Rao’s battery, [ii] the minimal assessment of cognitive function in MS [MACFIMS], and [iii] the Brief International Cognitive Assessment for Multiple Sclerosis [BICAMS; ISLAS], they are used especially in clinical trials. In clinical practice, SDMT has recently become the most used psychometric and adopted as screening test in MS, mainly due to its ease of administration, predictive validity, sensitivity and specificity. Also, it is well correlated with significant magnetic resonance imaging [MRI] measures, including brain atrophy, total lesion burden and microstructural pathology (12). Transient reduction of SDMT score was also used in defining cognitive relapses (13). Pardini et al. (13) proposed a definition of the isolated cognitive relapse [ICR] as a transient significant cognitive decline [reduction of SDMT≤4 points] associated with the presence of a gadolinium enhancing lesion on brain MRI (13).

Another important issue to consider is the subjective vs. objective cognitive complaints in MS patients. Therefore, appropriate cognitive testing in all MS subjects should be done on a regular basis. Moreover, we suggest cognitive functioning should be formally included in all MS treatment trials as a secondary outcome.

Cognitive functioning is not used as a standard primary outcome measure in assessing treatment efficacy. However, it should be noted that approximately 50% of patients achieving a classically defined “no evidence of disease activity” [NEDA-3, defined as absence of relapses, disability worsening, and MRI activity] after 2-years of follow-up had a noticeable deterioration in at least 2 cognitive domains (14). Moreover, 25% of patients had a meaningful cognitive decline, defined as a decrease of at least 4 points on SDMT, which was associated with a deterioration of the employment status (14).

### 1.2. Is brain atrophy a relevant surrogate marker of cognitive dysfunction in clinical trials?

Brain atrophy accumulates over the disease course and is a known correlate of current and future cognitive decline in MS patients. Patients with more severe structural damage at baseline are more prone to suffer from cognitive decline, which in early RRMS is predicted mainly by white matter integrity damage, while in late RRMS and progressive MS is predicted most accurately by cortical atrophy (15). However, the studies that looked for a relevant radiological predictor of cognitive decline reveal conflicting results. Uher et al. (16) found that the risk of confirmed cognitive decline over the 2-year follow-up was greater in patients with a high baseline T2 lesion volume and more pronounced baseline brain atrophy, measured as low brain parenchymal fraction [BPF] (16). In another study by Papathanasiou et al. (17) neuropsychological measures had a strong correlation with all MRI atrophy measures [third ventricle width, thalamic and corpus callosum atrophy] and weak or moderate one with total lesion volume. In their study the thalamic area was the most sensitive predictor of memory and psychomotor speed deficits (17).

Significant atrophy of the whole brain, the cortical gray matter [GM], hippocampus, deep GM nuclei, and the white matter [WM] was found in patients with MS-related cognitive impairment versus those who were cognitively preserved, despite similar levels of physical disability (18).

More recent studies have demonstrated a strong association between brain atrophy and cognition, focusing on correlations between specific cognitive tests and characteristic pattern of radiological abnormalities, i.e., SDMT with WM microstructural damage, or reduced PASAT performance with the atrophy of several gray matter regions, i.e., bilateral thalamus putamen and caudate nucleus (19). Our team found associations between regional black hole volumes and several cognitive functions (20). We also managed to identify a distinct regional brain atrophy pattern in multiple sclerosis, as compared with one of the MS mimickers, namely neuropsychiatric systemic lupus erythematosus (21) and this MS-specific pattern of global and subcortical gray matter atrophy correlated with cognitive impairment (22).

The obvious predictors of deteriorating cognitive performance include progressing cortical atrophy, older age and higher EDSS. On the contrary, higher cognitive reserve [CR] in individuals with MS may mediate this process, with higher CR predicting better performance on neuropsychological tests, independent of brain atrophy (23). The cognitive reserve is a phenomenon consisting of years of education, occupational status and all leisure activities that expand our cognitive abilities, e.g., social activities or reading books. In order to improve this protecting, intellectual enrichment, it is recommended to be engaged in leisure activities that require, among many, attention, memory, and planning (2).

### 1.3. Do disease-modifying therapies impact cognitive functions in multiple sclerosis?

Early treatment with disease-modifying therapies [DMTs] should be started immediately after the diagnosis, as it has been proven to decrease the risk of disability and delay conversion to secondary progressive MS (24) Consequently, it also appears to be the possible therapeutic intervention to prevent or delay the development of cognitive disability. DMTs’ beneficial effects on cognition may thus depend on the anti-inflammatory properties of the immune therapies. In the long-term, the protective effect against tissue damage may result not only from preventing the accumulation of the new inflammatory-demyelinating lesions, but also from preventing the progression of brain atrophy (25).

Brain atrophy seems to be the indirect way to assess DMTs’ impact on cognitive decline, especially since some cognitive domains, like information processing speed, are closely associated with global brain atrophy (26).

Another aspect of DMT action could be the promotion of neurotrophic factors production, which has been associated with the use of, i.e., beta-interferons or fingolimod in MS patients (27, 28). In RRMS, Brain-Derived Neurotrophic Factor [BDNF] and beta-Nerve Growth Factor [beta-NGF] are strongly linked to cognitive performance and may exert a neuroprotective role (29, 30). Another neurotrophic factor, namely neurotrophin-3 [NT-3] has been linked to brain atrophy (31).

The aim of the study was to review the evidence regarding how DMTs affect cognitive functions.

### 1.4. Search strategy

In the present clinical review, we included 44 articles written in English and reporting on the adult (>90% studies) and pediatric MS population, focusing on cognitive functions as the outcome of DMTs, 39 studies were peer-reviewed and 5 were abstracts.

We included all DMTs currently approved by FDA in US and EMA in United Europe: interferon beta-1a intra-muscular, interferon beta-1a subcutaneous, interferon beta-1b, peginterferon beta-1a, glatiramer acetate, natalizumab, ocrelizumab, alemtuzumab, ofatumumab, dimethyl fumarate, teriflunomide, fingolimod, cladribine, siponimod and ozanimod. We excluded interventions involving combination and nonpharmacological treatments.

Relevant studies were identified with the use of PubMed, and the European Committee for Treatment and Research in Multiple Sclerosis [ECTRIMS] Library, using the words: “cognition,” “cognitive deficit“, “cognitive outcome“‚ “SDMT” ‚” PASAT,” “DMT” (Table 1). We included double-blind Phase III or IV randomized controlled trials (RCTs), in which any of the DMTs was compared as monotherapy with placebo or another active drug for the treatment of RRMS, or SPMS (siponimod), with regards to neurocognitive functions, including cognitive processing speed, working memory and verbal learning. The neurocognitive domains were examined with the use of the following tests: Paced Auditory Serial Addition Test (PASAT), Symbol Digit Modalities Test (SDMT), The Brief Repeatable Battery (BRB) of Neuropsychological Tests, Brief NP Battery, Stroop Test, Wechsler Memory Scale. The minimum duration of treatment was 6 months. We also included data from real-world and observational open-label studies. Preclinical studies, retrospective studies, case reports, reviews, commentaries, and letters were excluded.

**Table 1:** Summary of the most important studies on cognitive functions and DMTs.

Authors	Year of publication	DMT	No of subjects	Test	DMT efficacy	Follow-up
Fischer et al.	2000	INFβ-1a im/placebo	83/83	Brief Np. Battery	Yes	2 y
Patti et al.	2013	INFβ-1a sc, COGIMUS Study	201	BRB, Stroop Test	Yes	5 y
Mori et al.	2012	INFβ-1a sc	80	PASAT	Yes	2 y
Benesova et al.	2017	INFβ-1a sc, SKORE Study	300	PASAT	Yes	2 y
Penner et al.	2012	INFβ-1b, BENEFIT Study	468	PASAT	Yes	5 y
Kappos et al.	2016	INFβ-1b, BENEFIT Study	278	PASAT	Yes	11 y
Barak et al.	2002	INFβ-1b/placebo	18 /23	BRB tests	Yes	1 y
Lacy et al.	2013	INFβ-1b	16	Wechsler Memory Scale, Stroop tasks	Yes	16 y
Weinstein et al.	2002	GA/placebo	125 /126	BRB	No	2 y
Ziemsen et al	2014	GA, COPTIMIZE Study	672	PASAT	Yes	2 y
Ziemsen et al.	2016	GA, QualiCOP	754	PASAT, MUSIC	Yes	2 y
Cinar et al.	2017	INFβ-1a sc/INFβ-1b/GA	53/52/56	BICAMS	Yes	1 y
Gartner et al.	2017	INFβ-1b, BETAPAEDIC Study	68	Wechsler Scale, Raven Matrices	Yes	2 y
Coyle et al.	2018	Teriflunomide,TERI-PRO	100	SDMT	Yes	48 weeks
Wuerfel et al.	2022	Teriflunomide/placebo, TEMSO	358/363	PASAT	Yes	2 y
Giovannoni et al.	2016	DMF/placebo DEFINE, CONFIRM	769 / 771	PASAT	Yes	96 weeks
Amato et al.	2020	DMF	217	BRB, Stroop tes	Yes	2 y
Kappos et al.; Cohen et al.	2016	Fingolimod/placebo freedoms, transforms studies	783/773	PASAT	Yes	2 y
Ozakbas et al.	2016	Fingolimod	96	SDMT, BVMTR, CVLT2	Yes	6 months
Barak et al.	2019	Fingolimod	29	Mindstream Computerized Global Assessment Battery	Yes	1 y
Cree et al.	2018	Fingolimod/injectable, PREFERMS Study	433/428	SDMT	No	48 weeks
Comi et al.	2017	Fingolimod/INFβ-1b, GOLDEN Study	106/51	Rao, BRB	Yes	18 months
Schulze et al.	2021	Fingolimod, PANGAEA Study	2,428	SDMT	Yes	2 y
Weinstock-Guttman	2012	Natalizumab/Placebo,AFFIRM Study	627/315	PASAT	Yes	2 y
Perumal et al.	2019	Natalizumab, STRIVE Study	222	SDMT	Yes	2 y
Wilken et al.	2013	Natalizuamb, ENER-G Study	89	ANAM	Yes	48 weeks
Giovannoni et al.	2017	Alemtuzumab/ INFβ-1a, CARE-MS Study	426/202	PASAT	Yes	2 y
Cohan et al.	2020	Ocrelizumab/INFβ-1a, OPERA I, II Studies	827/829	SDMT	Yes	96 weeks
Giovannoni	2021	Siponimod/placebo, EXTEND Core Study	903/427	SDMT	Yes	5 y
Benedict	2022	ofatumumab/teriflunomide ASCLEPIOS I/II	492/468	SDMT	Yes	2 y

BRB, Brief Repeatable Battery; MUSIC, Multiple Sclerosis Inventory Cognition Scale.

Table 1 contains the summary of the most important studies on cognitive functions and DMTs.

Table 2 contains description of the psychological tests mentioned in the review.

**Table 2:** Description of the psychological tests mentioned in the review.

Name of test	Abbreviation	Cognitive domains
Paced auditory serial addition test	PASAT	information processing speed and flexibility, calculation ability
Symbol digit modalities test	SDMT	processing speed, working memory, and learning
Brief repeatable battery of neuropsychological tests for multiple sclerosis Rao test	BRB-N	verbal memory and delayed recall, visuospatial memory and delayed recall, sustained attention and concertation, semantic memory
Multiple sclerosis inventory of cognition	MUSIC	attention including susceptibility to interference, working memory, flexibility, long-term memory
California verbal learning test ver. 2	CVLT 2	episodic verbal learning and memory
Brief visuospatial memory test–revised	BVMTR	visual memory, immediate visual recall
Brief international cognitive assessment for multiple sclerosis	BICAMS	processing speed, working memory, and learning, immediate visual recall
Wechsler memory scale	Wechsler	verbal, visual, working, and recognition memory
Stroop test	Stroop	selective attention capacity and skills, processing speed, executive skills
Automated neuropsychological assessment metrics	ANAM	attention, concentration, reaction time, memory, processing speed, decision-making

## 2. The impact of disease modifying therapies on cognitive functions

### 2.1. Beta-interferons and glatiramer acetate

Among all DMTs, platform therapies [beta-interferons, IFN, namely IFN beta-1a and IFN beta-1b, and glatiramer acetate, GA] have provided most studies with the longest follow-up data.

The phase III study with IFN beta -1a administered intramuscularly once a week evaluated the effect of the active compound on cognitive functions, by assessing 166 patients [83 on IFN and 83 on placebo] with the Brief NP Battery (32). In a 2-year period of IFN beta- 1a treatment performance measures of information processing and memory significantly improved relative to placebo, but no treatment effect was evident with regards to verbal abilities and attention span domains.

The COGIMUS study [including 201 patients] confirmed that IFN beta-1a [44 ug dose s.c. three times a week] stabilizes or even delays cognitive impairment over a 5-year period in most patients. The proportion of patients with cognitive impairment was 18% at baseline and 22.6% at year 5, which was significantly lower than the expected deterioration without treatment. Interestingly, the protective effect was greater in women than in men (33).

Mori et al. (34) divided a group of 80 treatment-naive MS patients who were to start IFN beta-1a s.c. treatment, into 2 subgroups depending on their radiological activity on baseline MRI (34). Patients who had gadolinium-enhancing lesions [Gd+] performed worse on the PASAT task compared with patients without active lesions. Both groups were similar with regard to disease duration, age, EDSS score and relapse rate. During 24 months of follow-up, the PASAT score improved in baseline Gd + patients and was stable in the baseline Gd- group. This study is particularly valuable because there are only a few studies concerning patients with isolated cognitive relapses [when gadolinium enhancing lesions are otherwise asymptomatic].

Another study concerning patients treated with IFN beta -1a s.c. was the SKORE observational study, with 300 patients enrolled. Patients were randomized into two sub-groups receiving two different doses of IFN [22 μg and 44 μg, respectively] (35). At all follow-up time points, the average cognitive performance improved. The proportion of patients with an increased or stable PASAT score vs. baseline was 57.7% at 6 months, 68.0% at 12 months and 61.4% at 24 months.

The BENEFIT study, including 468 patients with clinically isolated syndrome [CIS], revealed that improvement in PASAT-3 score from baseline to year 2 was greater for IFN beta-1b than for the placebo group. PASAT-3 is the 3 s-interstimulus interval and is one of the 3 tests included in the Multiple Sclerosis Functional Composite (MSFC), interstimulus intervals may be shorter e.g.2 s in other versions of PASAT. The treatment effect was maintained at year 5 and cognitive improvement was significantly more pronounced among patients that were treated early (36). After 11 years, patients from the BENEFIT trial were reassessed. The early-treatment group still had a better PASAT-3 score, and both groups were stable. These findings further confirm the conclusion that beta-interferons effectively protect from cognitive impairment (37), although the potential practice effect on the PASAT task should be acknowledged. Moreover, the BENEFIT population was generally affected with a mild disease course in both groups.

Other studies, based on the real- world data, usually enrolled small patient populations.

Barak et al. (38) confirmed that 1-year of treatment with IFN beta-1b had a positive effect on cognition measured with the BRB-N (38). The study was limited with a small sample size. It compared 18 MS patients treated with IFN beta-1b with 23 untreated subjects. The patients in the control group deteriorated in 3 out of 5 domains that were tested, while those that were treated improved in 2 domains, especially attention, concentration, and visual–spatial learning.

Lacy et al. (39) revealed a 16-years long effect of IFN beta-1b treatment on cognitive functioning, but again on a small sample. Sixteen IFN beta-1b treated patients remained relatively stable in their cognitive performance, which would not have been expected in the natural course of MS (39).

The first randomized, placebo-controlled study with glatiramer acetate, including 251 patients [125 on glatiramer and 126 on placebo], found no effect of GA treatment on the course of cognitive impairment. There were no differences in BRB-N scores at 24 months. Importantly, from the standpoint of the natural disease course, there was no measurable decline in cognitive functioning in either of the groups over the 2-year period. The authors speculated this was the beneficial effect from extra care and supportive social contact for all patients (40). After 2 years the participants were moved to the open-label extension study. Mean scores of memory and semantic retrieval tests did not change significantly, but both groups declined in attention, and the relapse rate during the first 2 years of the trial was a predictor of the cognitive decline (41).

The COPTIMIZE trial assessed patients after switching to GA from another therapy [>95% patients switched from interferons]. After 2 years patients improved PASAT scores by a mean 4.29 ± 9.28 (p,0.0001) (42). It would be more credible should there be a control group with other treatments or no treatment at all to verify if this improvement was not only due to the practice effect. However, this is a common limitation for other trials and studies. The improvement was observed in both, patients who were switched because of adverse events [AEs], and those who were switched for the lack of efficacy. However, the final scores were higher in the AEs group, which could be explained by the decreased fatigue following beta-interferons withdrawal, as the patients no longer experienced the flu-like side-effects, namely fever, fatigue, muscle pain, and headache.

The QualiCOP was an observational study with patients treated with GA, including subjects that were treatment-naive and those who were previously treated with GA (43). Cognitive outcome was measured with the PASAT and Multiple Sclerosis Inventory Cognition scale [MUSIC]. After 24-months follow-up patients improved significantly in both tests.

The efficacy of all injectable therapies seems to be similar. Cinar et al. (44) compared patients on 3 different injectables: IFN beta-1a s.c., IFN beta-1b s.c. and GA. A total number of 161 newly diagnosed patients were monitored with the use of BiCAMS for a period of 1 year (44). At baseline, the mean scores for all 3 cognitive tests were significantly higher in the control group than in the MS group. At month 12, all three scores improved in all MS groups compared with baseline, and there were no significant differences between the groups.

Of note, there are only few studies assessing cognitive functions in the pediatric MS population. In the BETAPAEDIC study, 68 treatment-naive patients who started IFN beta-1b were stable during 2 years of observation in the scores they obtained on Wechsler Intelligence Scale for Children, Raven’s Standard Progressive Matrices, the d2 Test of Attention and the Beery-Buktenica Developmental Test of Visual-Motor Integration (45). However, early administration of highly effective treatment in pediatric MS may better protect from cognitive decline, as patients who were escalated to natalizumab or fingolimod had cognitive performance preserved or ameliorated, while higher impairment was more prominent in those who remained on the first-line platform therapy. The results obviously need to be interpreted with caution, as they included only 19 participants (46).

### 2.2. Teriflunomide

The Teri-PRO study was a phase 4, real-world study that enrolled patients with RRMS who switched to teriflunomide from another DMT and received teriflunomide for 48 weeks. The SDMT scores were stable over that 48-week study period. Similarly, cognitive impairment, as recorded by patients on the cognitive domain of the MSPS [Multiple Sclerosis Performance Scale], also remained stable (47).

Importantly, there is a clearly recognized association between brain volume loss [BVL] and long-term accumulation of cognitive disability in the pivotal TEMSO study. Teriflunomide slowed BVL significantly compared to placebo [median BVL from baseline to year 2 was 1.29% for placebo and 0.90% for teriflunomide], which could suggest a neuroprotective role of teriflunomide (48). Patients who received 14 mg teriflunomide early on in the 2-year study experienced significant improvement in processing speed domain, compared to those who were randomized to placebo in the core study part, and benefits were extended for up to 5 years (49). This underscores the important role of early treatment initiation in the MS population.

### 2.3. Dimethyl fumarate

The DEFINE and CONFIRM trials were designed to compare delayed-release dimethyl fumarate [DMF] with placebo or glatiramer acetate, respectively (50). Brain atrophy analysis revealed 30% reduction in the percentage brain volume change [PBVC] from 6 months to 2 years in patients treated with DMF compared to those treated with placebo (51).

In the observational Italian study that recruited over 200 subjects the effects of DMF treatment on cognition were assessed with the use of BRB-N and Stroop tests. Cognitive impairment at baseline was reported in 22.6% of patients. At 2 years, only 44.1% worsened, and 55.9% did not. However, dataset was incomplete as only 69.3% patients with cognitive impairment at baseline completed the study (52).

### 2.4. Fingolimod

Most data from clinical trials with fingolimod provide indirect proof of its impact on cognition. This is mostly related to brain atrophy reduction in MS patients. FREEDOMS and TRANSFORMS studies indicate that the onset of action on BVL and cognition commenced early, within 3 to 6 months of treatment initiation. Fingolimod reduced brain atrophy and improved PASAT scores compared to placebo and IFN beta-1a [FREEDOMS and TRANSFORMS studies, respectively] (53, 54).

The post-hoc analysis of data from FREEDOMS revealed that fingolimod significantly improved PASAT scores versus placebo, which was regardless of baseline cognitive status, and the effect was sustained for up to 120 months (55).

In a trial reported by Ozakbas et al. (56) which included 96 patients and 98 healthy controls, a significant improvement in SDMT, CVLT2 and BVMTR scores was observed 6 months since fingolimod initiation (56).

In a smaller study after 1 year of fingolimod treatment 29 RRMS patients were cognitively stable (57). The participants underwent a comprehensive cognitive assessment with the use of the Mindstream Computerized Global Assessment Battery, measuring verbal and non-verbal memory, executive function, visual spatial perception, verbal function, attention, information processing speed and motor skills (57).

There are few studies comparing fingolimod with another drug with regards to its influence on cognition. Utz et al. (58) compared 33 patients treated with fingolimod or natalizumab using 8 neuropsychological tests. After 1 year follow-up,75% of patients were cognitively stable, and there was no difference between the two drugs (58).

A 48-week PREFERMS study compared 861 patients treated with either fingolimod or injectable DMTs [IFN beta-1a/b, glatiramer acetate]. The brain volume loss was less pronounced with fingolimod than with injectables, but no difference in SDMT scores was noted (59).

The GOLDEN study evaluated the effects of fingolimod and IFN beta-1b on cognitive impairment progression. At month 18, both groups showed improvement in all the measured cognitive parameters, as assessed with the use of Rao’s Brief Repeatable Battery and Delis–Kaplan Executive Function System test (60).

PANGAEA 2.0 is an ongoing real-world study assessing patients switching to fingolimod from other DMTs. In the present interim analysis of 2,428 patients, after 2 years all patients on fingolimod were found to have improved their SDMT scores, with the highest improvement observed in patients without any previous DMTs or with one DMT prior to fingolimod use (61). This analysis included patients switched from injectables [beta-interferons or glatiramer acetate] and oral DMTs [dimethyl fumarate or teriflunomide].

### 2.5. New sphingosine 1-phosphate receptor modulators

Siponimod is a novel S1P1 and S1P5 receptor modulator, which has been approved for secondary progressive MS. In the preclinical models it was shown to promote remyelination, as S1P receptors are also expressed by oligodendrocytes, neurons, microglia and astrocytes (62). In the EXPAND Core Study siponimod significantly reduced the risk of a meaningful worsening in the cognitive processing speed [defined as ≥4point decline in the SDMT score] versus placebo (63).

The effect was sustained for up to 5 years of observation. Interestingly, in the active SPMS subgroup the benefits were more pronounced, which invariably supports the earlier treatment initiation.

The SUNBEAM study compared ozanimod versus intramuscular IFN beta-1a, and the improvement on the SDMT score at month 12 was greater for ozanimod-treated patients, although the effect size was trivial, and the overall composite MSFC score was not significantly different between both groups (64). We did not find any data regarding cognition for ponesimod.

### 2.6. Natalizumab

In the AFFIRM pivotal study of 856 patients, the percentage of patients with confirmed progression of cognitive deficit at 2 years was 7% in the natalizumab group vs. 12% in the placebo group, as assessed by the PASAT test [*p* = 0.013] (65).

STRIVE was an observational open-label study of 222 natalizumab-treated patients, where additional secondary endpoints included changes in cognition. A clinically significant improvement [an increase in SDMT score of ≥4 points] was observed in 41.9% of patients at year 1, and 49.4% of patients at year 2, which was associated with the general improvement of the work capacity (66).

Gudesblatt et al. (67) measured changes in cognition with the use of NeuroTrax computerized battery of tests in 57 patients who were treated with natalizumab for at least 2 years (67). The percentage of patients experiencing a significant improvement in the Global Cognitive Score increased from 21.6% at year 1 to 32.7% at year 2, regardless of the treatment-naïve or previously treated status. The greatest relative reduction of the deficit was observed in the attention and information processing speed domains.

Jacques et al. (68) demonstrated the long-term impact of natalizumab on cognition. Sixty-two patients [divided into two groups: treated longer and shorter than 2 years] were assessed with SDMT and CogState battery before every natalizumab infusion over a 24-month period. No patient in either group showed evidence of sustained cognitive deterioration. Moreover, in both groups, significant improvement in the mean scores of executive functions, verbal memory and working memory was observed (68).

Kunkel et al. (69) investigated a group of 51 natalizumab-treated patients and showed improvements in responsiveness, divided attention and information processing speed, although the percentage of patients suffering from fatigue increased from 55% at baseline to 61% in the second year (69).

The ENER-G study [89 patients] showed that cognitive performance in Automated Neuropsychological Assessment Metrics [ANAM] tests improved or remained stable up to 48 weeks after initiation of natalizumab. Fatigue was also reduced on therapy (70).

There are fewer studies where natalizumab is compared to other DMTs with regards to cognition. Sundgren et al. (71) demonstrated that natalizumab treatment for 1 year did not significantly improve cognitive functioning in RRMS patients compared to control patients on stable first-line DMT [IFN beta -1a i.m.]. In both groups participants with lower baseline scores had a significantly greater improvement (71). Other small sample studies by Rorsman et al. (72) [34 patients], and Portaccio et al. (73) [16 patients] found that natalizumab was more effective than first line therapy in reducing cognitive deterioration.

In an interesting study concerning NTZ withdrawal, patients who stopped NTZ treatment [because of PML risk] were compared with those who continued therapy. Neuropsychological assessment [BRB and the Stroop test] after 1 year revealed that 63.3% patients discontinuing NTZ presented with a cognitive worsening, whereas in the continuers’ group it was only 7.1% (74). Preziosa et al. (75) compared 30 patients on NTZ with 25 on fingolimod therapy, at month 24 both drugs improving the MSFC score (75).

With the schedule of Extended Interval Dosing [EID] of NTZ more and more frequently used worldwide, McManus et al. (76) assessed the impact of EID on cognitive parameters. In a group of 34 patients on EID-NTZ schedule, improved cognitive Z scores after 28 months of treatment were observed, especially in memory, attention and executive function (76).

Natalizumab seems to preserve cognition also in a pediatric-onset MS, where patients are at a high risk of developing cognitive impairment in their adulthood. In a group of 20 treatment-naive patients started on natalizumab, the SDMT score improved in 13 of them after 24 months of treatment and declined in only 2 patients (77).

### 2.7. Alemtuzumab

In CARE-MS II study of alemtuzumab, annual BVL throughout years 3–5 and cumulative BVL over 5 years was smaller in patients who received alemtuzumab compared to IFN beta-1a s.c,. which might indicate neuroprotective effects (78). However, there was no significant difference in the PASAT scores between both groups (79).

In a smaller study, Riepl et al. (80) assessed 21 patients treated with alemtuzumab. After 15 months, overall cognitive functioning of patients remained stable or improved. The proportion of patients that showed deficits in≥3 tests was reduced from 24% at baseline to 14% at follow-up, especially in the processing speed domain (80). The authors explored whether cognitive change from baseline to follow-up was dependent on clinical changes [EDSS, T2 lesion load, relapse rate], but surprisingly none of them was a significant predictor of cognitive function.

In a small group of 17 alemtuzumab-treated patients Hvid et al. confirmed the effectiveness of the drug on cognitive functioning, observing the improvement in the selective reminding test and SDMT after 24-month of therapy (81).

The trend showing a link between the improvement of cognitive functions and higher potency of immunotherapy was also confirmed by the study where 19 patients receiving autologous hematopoietic stem cell transplantation [aHSCT] were compared with 21 patients receiving alemtuzumab. The wide battery of neuropsychological tests was used i.e., SDMT, Verbal Learning and Memory Test, test of attention [TAP] (82). Patients receiving aHSCT showed improved cognitive functioning [mean follow-up 58.8 months], while alemtuzmab-treated subjects deteriorated in all the tested domains [mean follow-up of 27.6 months].

### 2.8. Ocrelizumab

The OPERA I and OPERA II studies showed that ocrelizumab use was associated with a statistically significant improvement in SDMT scores over 96 weeks, compared with IFN beta-1a s.c (83). Also, ocrelizumab-treated patients had a significantly lower risk of developing sustained SDMT decline over 12 and 24 weeks. Such trend was also observed in subgroups of patients with moderate cognitive impairment at baseline.

Patients on ocrelizumab had 57% [OPERA I] and 64% [OPERA II] lower number of new hypointense lesions on T1-weighted MRI. However, the differences in the percentage of brain-volume loss from week 24 to week 96 were non-confirmatory in the OPERA I and non-significant in the OPERA II studies (84).

### 2.9. Ofatumumab

In the Phase 3 ASCLEPIOS I/II trials ofatumumab significantly improved the SDMT scores from baseline to Month 24, more patients on ofatumumab had ≥4 point sustained improvement on SDMT versus teriflunomide (25% vs. 19.6%, *p* = 0.005) (85).

### 2.10. Cladribine

The CLARITY study confirmed the efficacy of cladribine in RRMS, but cognitive functioning was assessed only based on the 4 items from the SF-36 The Short Form 36 Health Survey is a 36-item, patient-reported survey of patient quality of life (86).

The CLADQoL is an ongoing prospective study where one of the secondary objectives is cognitive status assessed with SDMT. The final report is planned on December 2024.

## 3. Conclusion

As cognitive impairment in multiple sclerosis may be as devastating as physical disability, maintaining patients’ cognition or improving their cognitive deficits should naturally be our key therapeutic goal, besides reducing relapse rate and radiological activity. The stabilization of cognitive functions could be proposed as another outcome in the NEDA score (87), following atrophy normalization [NEDA-4] (88) and neurofilament levels stabilization.

Natural history studies in patients with MS suggest the rate of cognitive decline is approximately 5% per year (89). One of the studies that assessed a large contemporary cohort of patients on DMT with a long-lasting disease, with cognitive assessment after at least 10 years from disease onset, observed a much lower rate of cognitive impairment than previously reported in the pre-disease modifying treatment era (90). We may presume that DMTs have influenced cognition alongside other MS symptoms. However, there are many difficulties with verifying individual DMT’s potential therapeutic effect on cognition and comparing their effectiveness. Cognitive performance can be influenced by a number of factors, including cognitive reserve, fatigue, medications, infections, depression, comorbidities or cognitive rehabilitation. To date, most studies provide only indirect arguments for cognitive efficacy of DMTs, mainly by pointing to the reduction of brain atrophy rate. Cognitive impairment may start in different stages of the disease. Some patients may be protected longer by their cognitive reserve, and short-term [1 or 2 years] follow-up may not be enough to reveal cognitive decline and the therapeutic effects of DMTs. The time of follow-up cognitive assessment in MS patients should obviously be longer. However, in MS trials the 1–2 years follow-up is a pure reflection of the design of the pharmacoclinical studies. This is a clear limitation.

Early clinical trials of disease-modifying therapies did not include neuropsychological tests. Since cognition improved on validity, most of the current clinical trials use cognitive impairment as an outcome measure, although still only as secondary, tertiary, or exploratory outcomes.

Additionally, different neuropsychological tests were used across clinical trials, which makes it difficult to compare them in this aspect. Also, cognitive impairment is often characterized by overall performance based on several tests within a specific battery, with different patients impaired in different cognitive domains (90). As neuropsychological batteries are time-consuming, many studies have been conducted with the use of a single neuropsychological test. While SDMT seems to be a good, reliable [albeit simplified] screening test, PASAT test was mostly used in older studies and since then was shown to be burdened with the practice effect (91). PASAT measures attentional process as information processing speed, sustained attention but also working memory (91).

On the other hand, post-marketing studies usually have small sample sizes, short follow-up periods and are limited by the absence of the control groups.

Fortunately, neuropsychological assessment is now commonly incorporated into clinical trials, at times even as a primary outcome, like in the ENLIGHTEN trial with ozanimod, where the primary outcome is the increases in the SDMT score [estimated completion date 2025] (92). Since full neuropsychological testing is not feasible, a screening with SDMT has been gradually incorporated into routine clinical practice, at baseline in newly diagnosed patients and at regular follow-ups during the treatment period, but this is still far from the routine.

In summary, there is a line of evidence for cognitive benefits of DMTs, the following drugs are effective in preserving cognitive functions: beta-interferons, teriflunomide, dimethyl fumarate, siponimod, ozanimod, natalizumab, alemtuzumab, ofatumumab, ocrelizumab and cladribine.

Conflicting results concern glatiramer acetate and fngolimod, but more studies, especially in the real-world setting, are urgently needed. Highly effective therapies seem to be more effective in preventing cognitive decline. However, a recently published meta-analysis including 41 studies, while revealing a positive effect of DMTs on cognition, failed to show a statistically significant difference between platform and escalation therapies (93). The authors emphasized that some therapies were underrepresented or not taken into consideration at all, e.g., alemtuzumab, ocrelizumab, teriflunomide or cladribine. Even meta-analyzes collect and analyze data previously acquired from RCTs or RWD. In case of DMT’s effect on cognition good quality data is still lacking. Therefore, the value of such meta-analysis is also limited (94).

It is still a matter of debate whether cognitive impairment should be an argument to switching/escalating DMTs. Clinicians need more clues from clinical studies supporting such decisions. Also, if among contemporary treated population of MS only 50% of patients reach annual NEDA-3, is it feasible for the time being to expect NEDA-4, or even higher? One needs to consider that NEDA might not serve as an all-purpose goal of treatment. Also, there may be a group of patients, where minimizing cognitive impairment would be even more essential than reducing relapse rates or MRI activity.

**Figure 1:**
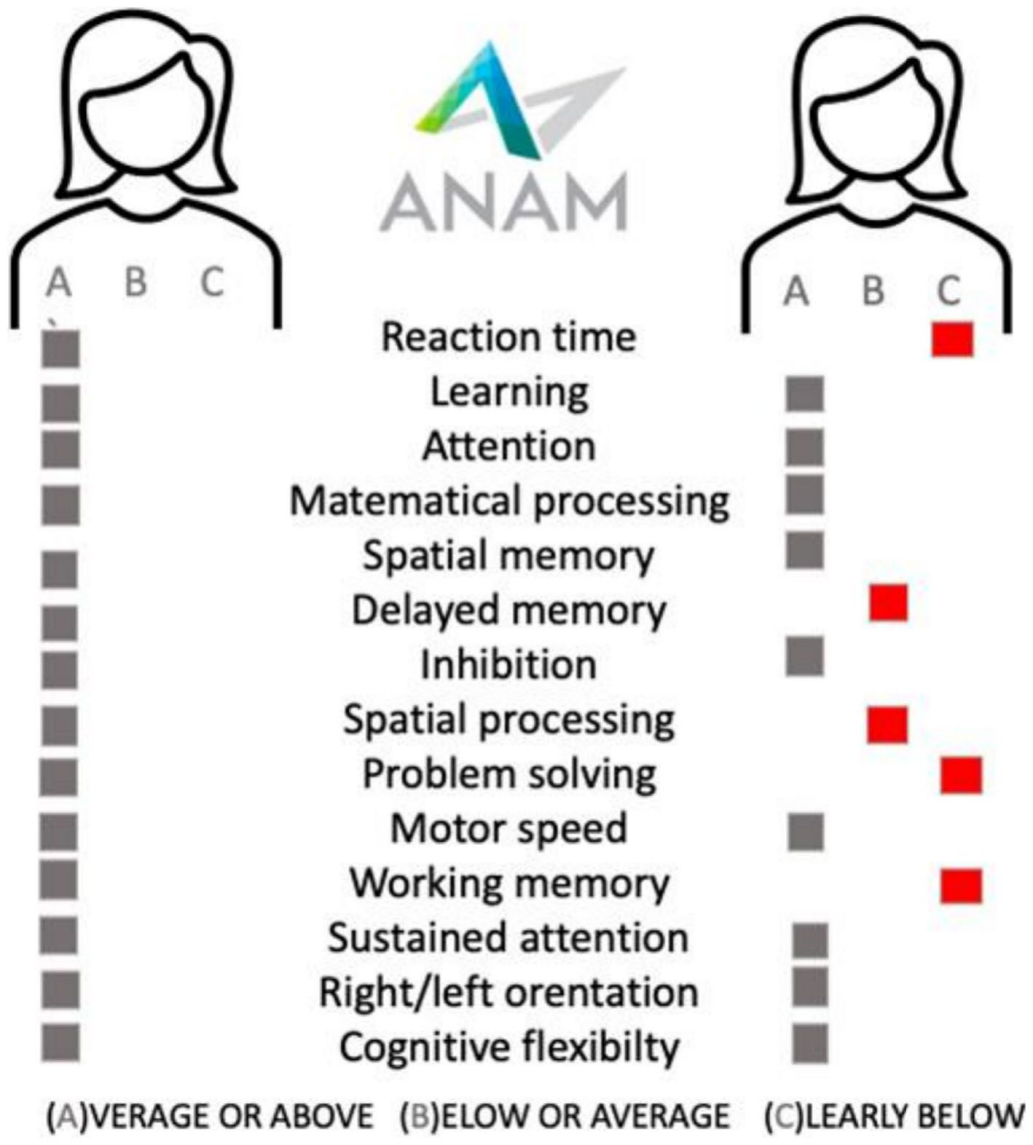
The examplary cognitive battery testing [ANAM, Automated Neuropaychological Assessment Metrics] results in sex, age, education, disease duration, treatment duration, EDSS, and demyelinating lesions volume matched two patients treated with beta-interferon.

Since” time is brain,” time is also preserved cognition. It is highly important to identify MS patients with cognitive impairment at baseline (Figure 1) or patients with a high risk of developing cognitive impairment, to make sure they promptly start effective therapy, and to propose a cognitive neurorehabilitation plan so that they maintain their quality of life for as long as possible.

## Author contributions

KK, WA, AK-Ł, and WK contributed to conception and design of the study. KK organized the database and wrote the first draft of the manuscript. KK, AK-Ł, and WA wrote sections of the manuscript. All authors contributed to manuscript revision, read, and approved the submitted version.

## Conflict of interest

The authors declare that the research was conducted in the absence of any commercial or financial relationships that could be construed as a potential conflict of interest.

## Publisher’s note

All claims expressed in this article are solely those of the authors and do not necessarily represent those of their affiliated organizations, or those of the publisher, the editors and the reviewers. Any product that may be evaluated in this article, or claim that may be made by its manufacturer, is not guaranteed or endorsed by the publisher.
